# Six-month supplementation with high dose coenzyme Q10 improves liver steatosis, endothelial, vascular and myocardial function in patients with metabolic-dysfunction associated steatotic liver disease: a randomized double-blind, placebo-controlled trial

**DOI:** 10.1186/s12933-024-02326-8

**Published:** 2024-07-10

**Authors:** Emmanouil Vrentzos, Ignatios Ikonomidis, George Pavlidis, Konstantinos Katogiannis, Emmanouil Korakas, Aikaterini Kountouri, Loukia Pliouta, Eleni Michalopoulou, Emilia Pelekanou, Dimitrios Boumpas, Vaia Lambadiari

**Affiliations:** 1 4th Department of Internal Medicine, Medical School, Attikon University Hospital, National and Kapodistrian University of Athens, Athens, Greece; 2 Laboratory of Preventive Cardiology and Echocardiography, 2nd Cardiology Department, Medical School, Attikon University Hospital, National and Kapodistrian University of Athens, Athens, Greece; 3 2nd Department of Internal Medicine, Research Unit and Diabetes Center, Medical School, Attikon University Hospital, National and Kapodistrian University of Athens, Athens, Greece

**Keywords:** Coenzyme Q10, MASLD, NAFLD, Liver steatosis, Cardiovascular diagnostic techniques

## Abstract

**Backround:**

Metabolic-dysfunction Associated Steatotic Liver Disease (MASLD) has been associated with increased cardiovascular risk. The aim of this Randomized Double-blind clinical Trial was to evaluate the effects of coenzyme-Q10 supplementation in patients with MASLD in terms of endothelial, vascular and myocardial function.

**Methods:**

Sixty patients with MASLD were randomized to receive daily 240 mg of coenzyme-Q10 or placebo. At baseline and at 6-months, the a)Perfused boundary region of sublingual vessels using the Sideview Darkfield imaging technique, b)pulse-wave-velocity, c)flow-mediated dilation of the brachial artery, d)left ventricular global longitudinal strain, e)coronary flow reserve of the left anterior descending coronary artery and f)controlled attenuation parameter for the quantification of liver steatosis were evaluated.

**Results:**

Six months post-treatment, patients under coenzyme-Q10 showed reduced Perfused boundary region (2.18 ± 0.23vs.2.29 ± 0.18 μm), pulse-wave-velocity (9.5 ± 2vs.10.2 ± 2.3 m/s), controlled attenuation parameter (280.9 ± 33.4vs.304.8 ± 37.4dB/m), and increased flow-mediated dilation (6.1 ± 3.8vs.4.3 ± 2.8%), global longitudinal strain (-19.6 ± 1.6vs.-18.8 ± 1.9%) and coronary flow reserve (3.1 ± 0.4vs.2.8 ± 0.4) compared to baseline (*p* < 0.05). The placebo group exhibited no improvement during the 6-month follow-up period (*p* > 0.05). In patients under coenzyme-Q10, the reduction in controlled attenuation parameter score was positively related to the reduction in Perfused boundary region and pulse wave velocity and reversely related to the increase in coronary flow reserve and flow-mediated dilation (*p* < 0.05 for all relations).

**Conclusions:**

Six-month treatment with high-dose coenzyme-Q10 reduces liver steatosis and improves endothelial, vascular and left ventricle myocardial function in patients with MASLD, demonstrating significant improvements in micro- and macro-vasculature function.

**Trial Registration:**

NCT05941910

## Background

Coenzyme Q10 (CoQ10) is a lipid-soluble quinone with a central benzoquinone ring, whose principal role in the cell is to participate in the electron transport chain in the inner mitochondrial membrane [1]. In addition, CoQ10 protects cellular membranes, both mitochondrial and extra-mitochondrial, against oxidative stress as a crucial lipid-soluble antioxidant. Both the oxidized (ubiquinone) and reduced (ubiquinol) forms of CoQ10 exist, and their constant conversion is necessary for the function of CoQ10 [2].

CoQ10 has been used to treat diseases that have oxidative stress involvement and excessive reactive oxygen species (ROS) production, such as cardiovascular disease (CVD) [3], dyslipidemia [4], hypertension [5] and endothelial dysfunction [6]. Along with CVD, the administration of CoQ10 has also been studied for chronic obstructive pulmonary diseases [7] and neurodegenerative disorders [8, 9].

Metabolic Dysfunction Associated Steatotic Liver Disease (MASLD), formerly known asnon-alcoholic fatty liver disease (NAFLD) is a hepatic manifestation of metabolic syndrome associated with obesity and increased CVD risk. This excess fat in the liver leads to an inhibition of insulin signaling and chronic inflammation which increases the risk of chronic liver diseases [10, 11]. Diverse investigations have confirmed the hypothesis that oxidative stress and ROS may play a role in the development of fibrosis [12]. In fact, it has been shown that MASLD patients have mitochondrial dysfunction and low levels of antioxidant defenses [13]. Despite the fact that MASLD has become one of the most significant and relevant chronic liver disorders worldwide, largely due to the global obesity epidemic, treatment options remain unclear. Recently, the U.S. Food and Drug Administration approved resmetirom for the treatment of adults with noncirrhotic non-alcoholic steatohepatitis with moderate to advanced liver fibrosis, to be used in conjunction with diet and exercise [14]. Given the high prevalence of MASLD in patients with diabetes mellitus (DM), antidiabetic drugs are frequently utilized for the clinical management of DM patients with MASLD. Most clinical studies on the use of glucagon-like peptide-1 receptor agonists have demonstrated resolution of liver steatosis and improvement in liver enzymes and metabolic parameters. Similarly, sodium-glucose co-transporter 2 inhibitors have shown improvements in liver steatosis and fibrosis, while pioglitazone has been associated with improved liver histology [15].

The purpose of the present prospective double-blind randomized clinical trial was to assess the effect of six-month supplementation of CoQ10 on endothelial, vascular and myocardial function in patients with MASLD.

## Methods

### Subjects

Adult patients (≥ 18 years of age) with Steatotic Liver Disease who had at least one extra cardiometabolic risk factor and without DM, who were referred to the hepatology and metabolic outpatient clinics of Attikon University Hospital of Athens, were prospectively enrolled in the study by the attending physicians (E.P. and V.L.). Steatotic Liver was confirmed by FibroScan® (Echosens, Paris, France). According to MASLD criteria [16], cardiometabolic risk factors are dyslipidemia (high density lipoprotein (HDL) < 40 mg/dL in men; < 50 mg/dL in women or triglycerides ≥ 150 mg/dL or drug treatment for elevated triglycerides and/or lipids), increased body weight (waist circumference ≥ 94 cm in men or ≥ 80 cm in women or body mass index (BMI) > 25), arterial hypertension (office brachial blood pressure ≥ 130/85 mmHg or use of antihypertensive) and dysglycemia (fasting serum glucose ≥ 100 mg/dL or two-hour post-load glucose levels ≥ 140 mg/dL or glycated hemoglobin (HbA1c) ≥ 5.7%). Exclusion criteria were the presence of DM, chronic kidney disease, heart failure, liver failure, pregnancy/breast feeding, active malignancy (receiving chemotherapy/immunotherapy), alcohol overconsumption (≥ 140 g/week for women or ≥ 210 for men)and anti-obesity medical therapy.

The investigation follows the guidelines set forth in the Helsinki Declaration. The Institutional Review Board of University General Hospital “Attikon” gave their approval to the study protocol (Approval number: 441/13-07-2022). Before receiving their written agreement to participate, all individuals were thoroughly informed about the study’s nature and goals. The study has been registered to ClinicalTrials.gov under ID number: NCT05941910.

### Study design and protocol

In this double-blind, placebo-controlled trial, all participants were randomly assigned in a 1:1 ratio to receive 2 tablets of CoQ10 daily (full tablet components: 120 mg CoQ10, 90 mg Choline, 100 mg Milk Thistle, 50 mg Cynara, 30 µg Selenium, 80 mg Vitamin C, 1.1 mg Vitamin B1, 1.4 mg Vitamin B6, 5 µg Vitamin B12) or 2 tablets of placebo daily (tablet component: Microcrystalline cellulose) for 6 months. The assignment to a treatment regimen was performed by an attending physician (E.V.) using a table of random numbers as produced from the online randomization software http://www.graphpad.com/quickcalcs/index.cfm. All patients received dietary instructions based on Mediterranean diet, and motivation to engage in at least 150 min of moderate exercise per week. Each participant underwent two outpatient visits: one during the baseline visit and the other six months later. Moreover, monthly phone calls were systematically conducted with every study participant. The primary objectives encompassed the provision of motivational support and the confirmation of steadfast adherence to the prescribed study protocol.

In both visits, blood samples were collected for routine analyses and examination of HbA1c, total cholesterol (TC), triglycerides (TG), HDL, low-density lipoprotein (LDL) cholesterol and C-reactive protein (CRP). Furthermore, a standard 75-gr oral glucose tolerance test (OGTT) test was performed to exclude DM. Venous blood was sampled at 0, 60 and 120 min after glucose load to determine plasma glucose and serum insulin. All patients underwent clinical, vascular, and echocardiography evaluation with liver fat estimation in both visits. The above examinations were carried out by experienced physicians who were blinded for the history of the patients.

### Outcomes

The primary end point was changes in controlled attenuation parameter (CAP) values and Left Ventricular Global Longitudinal Strain (LV GLS) after six-month administration of CoQ10 compared to placebo.

Secondary end points were changes inendothelial glycocalyx thickness, Pulse Wave Velocity (PWV), Flow-Mediated Dilation (FMD) of the brachial artery and Coronary Flow Reserve (CFR).

### Liver stiffness and CAP measurement

Transient elastography with FibroScan was utilized to evaluate CAP and liver stiffness. In order to get both the liver stiffness measurement and the CAP, the M probe was initially used. When the M probe malfunctioned, the XL probe designed for obese people was used. In each patient more than 10 reliable measurements were obtained. The following CAP cut-off values for liver steatosis (S) were derived from Kamali et al. study [17] as follows: (1) < 237 dB/m (S0, no steatosis), (2) 237.0-259.0dB/m (S1, mild steatosis), (3) 259.0-291.0 dB/m (S2, moderate steatosis), and (4) 291.0-400.0 dB/m (S3,severe steatosis). The cut-off values for fibrosis (F) were as follows: (1) < 5.5 kPa (F0, no fibrosis), (2) 5.5-8.0 kPa (F1, mild fibrosis), (3) 8.0–10.0 kPa (F2, moderate fibrosis), (4) 11.0–16.0 kPa (F3, severe fibrosis), and (5) > 16.0 kPa (F4, cirrhosis).

### Blood pressure measurement

Each patient sat quietly in a 23 °C room for 10 min. An automated digital oscillometric sphygmomanometer (TensioMed, Budapest, Hungary) was used to measure the brachial blood pressure and heart rate in the right arm. The mean result from two consecutive measurements separated by a 2-minute period was obtained and used in the analysis.

### Endothelial glycocalyx

Sidestream Dark Field imaging (Microscan, Glycocheck, Microvascular Health Solutions Inc, Salt Lake City, UT) was utilized to measure the perfused boundary region (PBR) of sublingual artery microvessels with diameters ranging from 5 to 25 μm. This technique allows for a quick and noninvasive measurement of endothelial glycocalyx thickness [18]. The PBR is the cell-poor layer formed on the surface of the vascular lumen by the separation of the flowing red blood cell column and plasma. A higher PBR value indicates that blood cells have penetrated deeper into the luminal region of the glycocalyx and represents a precise marker of decreased glycocalyx thickness. The measurement of glycocalyx thickness with specialized cameras takes 3 min and provides measurements of several sample locations (> 3000 vascular segments of sublingual microvessels). As a result, the European Society of Cardiology Working Group on Peripheral Circulation proposed this technique as a viable technique for assessing endothelium integrity [19].

### Central hemodynamics

The Complior tonometry (Alam Medical, Vincennes, France) was used to measure PWV (m/sec), augmentation index (AIx), central pulse pressure, and central systolic blood pressure. For PWV measurement the carotid and femoral waveforms were recorded using two non-invasive pressure sensors, and the space between the two artery sites was calculated using a tape measure. PWV values below 10 m/s were considered normal [20]. AIx was computed using the Eq. 100x(P2-P1)/PP, where P2 denoted the late backward systolic wave, P1 denoted the early forward systolic wave, and PP was the pulse pressure [21].

### Echocardiography

Studies were carried out utilizing a Vivid E95 ultrasound system (GE Medical Systems, Horten, Norway). Digital data were saved in an EchoPac GE 206 computer station. Two observers (I.I, K.K) blinded to clinical and laboratory data evaluated each study. Each subject had sufficient images for analysis.

#### Two-dimensional strain and strain rate analysis

Using the 17 LV segment model and specialized software (EchoPac PC 206; GE Healthcare, Horten, Norway), the LV GLS (%) from 2-dimensional echocardiogram images collected with a frame rate of 70/s to 80/s, from the apical 4-, 2-, and 3-chamber views were evaluated [21]. LV GLS is a sensitive marker for the early detection of LV systolic dysfunction, often identifying abnormalities before changes in left ventricular ejection fraction are detectable. Additionally, LV GLS offers incremental prognostic information across a range of clinical conditions, such as hypertrophic cardiomyopathy, coronary artery disease, and valvular heart disease, enhancing risk stratification and guiding clinical management. According to reports, the normal value for GLS is − 22.5 ± 2.7% [22, 23].

#### CFR measurement

Coronary flow velocities in the left anterior descending coronary artery were measured using a 7-MHz transducer and color-guided pulse-wave Doppler from long-axis apical projections [24]. The maximal velocity (CF-Vmax) of the diastolic component of the coronary flow wave (CF-VTI) were assessed at baseline and after adenosine infusion(140 mg x kg^−1^ x min^−1^) for 3 min [25]. CFR was computed as the ratio of hyperemic to resting CF-Vmax. The results of three cardiac cycles were averaged. The standard deviation (SD) of the differences between the first and second measurements was calculated and reported as a percentage of the average value to determine inter- and intra-observer variability.

### FMD measurement

The FMD of the brachial artery was evaluated and the results were reported as a percentage change in the arterial diameter during hyperemia compared to the baseline diameter. FMD represents a widely used test for assessing endothelial function because it is non-invasive, relatively simple to perform, and highly reproducible. The clinical significance of FMD lies in its ability to predict future cardiovascular events. Impaired FMD reflects the cumulative effect of various cardiovascular risk factors, such as hypertension, hyperlipidemia, diabetes, and smoking, on vascular function. It serves as an intermediate biomarker that can detect early endothelial dysfunction, which is a precursor to subclinical target organ damage and subsequent clinical events.The normal value for FMD is reported to be 8.2 ± 2.3% [26, 27].

### Statistical analysis

All analyses were performed with the Statistical Package for Social Sciences(IBM SPSS Statistics for Windows, Version 26.0. Armonk, NY, USA). Normally distributed continuous variables were shown as mean value ± standard deviation. Data with a non-gaussian distribution were expressed as median with interquartile range (first quartile–third quartile) and were analyzed after transformation into ranks. Differences in mean values for each of the measured markers were compared by Student’s t-testor Mann-Whitney U test, as appropriate. Parametric (Pearson r) and non-parametric (Spearman rho) correlation coefficients were used to examine cross-sectional associations. Analysis of variance (ANOVA) for repeated measurements was performed (a) for measurements of the examined markers at baseline and 6 months after treatment used as a within-subject factor and (b) for the effects of treatment (CoQ10 vs. placebo), as a between-subject factors. Age, sex, BMI, risk factors and medication were included as covariates in the model. The F and P values of the interaction between time of measurement of the examined markers and type of treatment were evaluated. Furthermore, the F and P values of the comparison between treatments were calculated. All statistical tests were two tailed and values of *p* < 0.05 were considered statistically significant.

We planned to study the percent change (Δ) of CAP after treatment from independent control (patients on CoQ10) and experimental subjects (patients on placebo) with one control per one experimental subject. In a pilot study of 10 patients treated with CoQ10 and 10 treated with placebo, the response within each group was normally distributed with standard deviation 34%. The true difference between patients under CoQ10 and those under placebo in the means of ΔCAP was 9%. Hence, we would need to study 30 patients treated with CoQ10 and 30 treated with placebo, to be able to reject the null hypothesis that the population means for ΔCAP after treatment of CoQ10 and placebo groups are equal with probability (power) 0.8 and type I error probability 0.05.

## Results

### Enrollment and patients

**Fig. 1:**
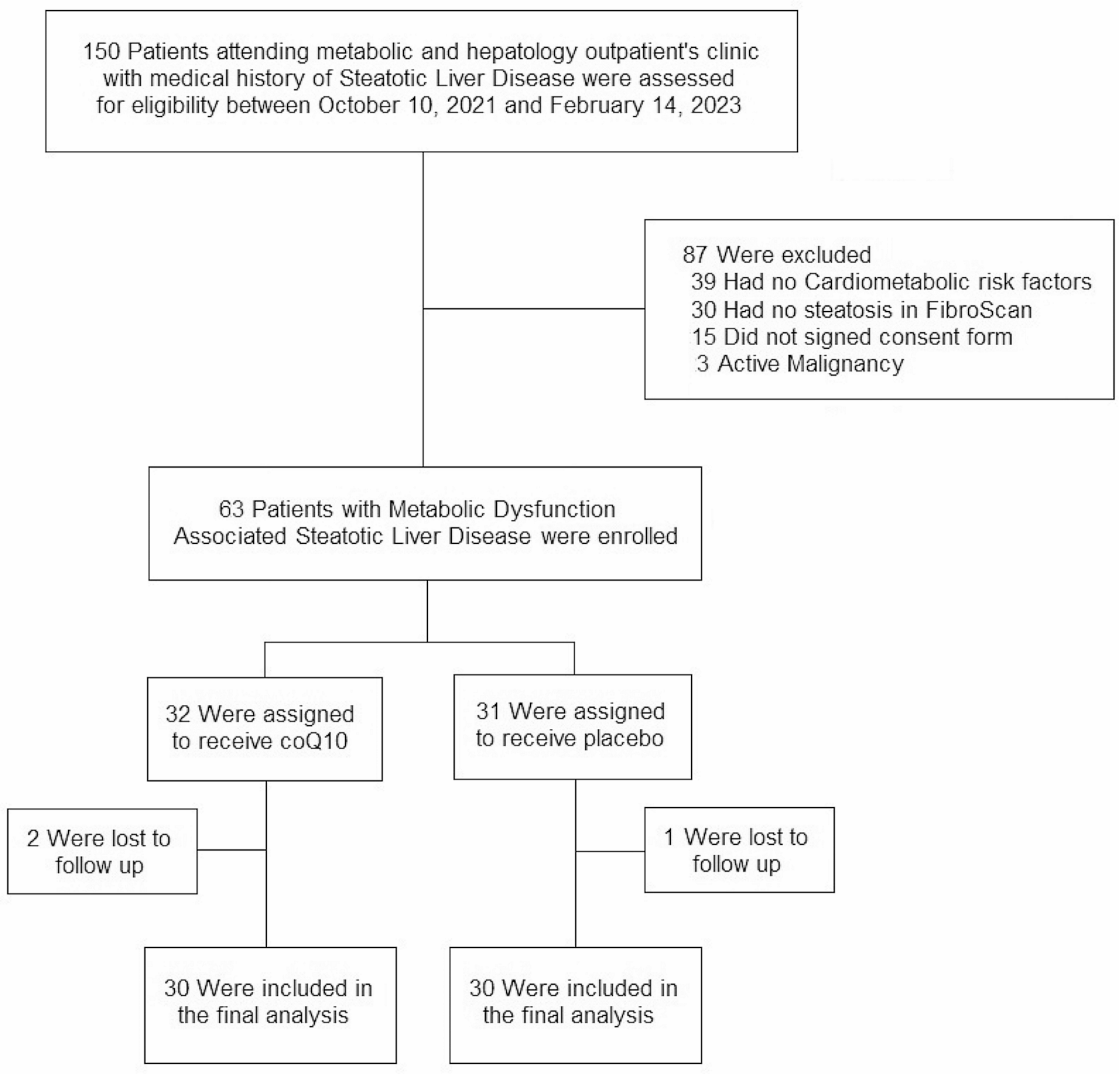
Screening, enrollment, randomization, and inclusion in analysis. CoQ10, coenzyme Q10

Enrollment began on October 19th, 2021 and ended on February 14th, 2023. A total of 63 patients were firstly enrolled (55% males, mean age 52 ± 10 years), 2 participants in the CoQ10 group and 1 participant in placebo group were lost to follow up. In the final analysis 30 patients in the CoQ10 group and 30 patients in the placebo group were included. No patients who were initially assigned to the active medication (Q10) or placebo had to switch to the alternate treatment throughout the study. Therefore, the results of the intention-to-treat analysis were identical to results of the per-protocol analysis for all the examined markers (Fig. 1).

Table 1 displays the study population’s baseline characteristics. At inclusion, all patients did not exhibit significant differences in regards to age, sex, fasting glucose, HbA1c, CRP, cardiovascular risk factors, and medications. During the trial period all participants continued their baseline medications.

**Table 1:** Demographic and clinical characteristics of the study population

	Total (*n* = 60)	CoQ10 (*n* = 30)	Placebo (*n* = 30)	*p*-value
Age (years)	52 ± 10	51 ± 10	53 ± 11	0.252
Sex (male), n (%)	33 (55)	17 (57)	16 (53)	0.813
Fasting glucose (mg/dL)	104 ± 14	101 ± 16	106 ± 17	0.224
HbA1c (%)	5.7 ± 0.6	5.7 ± 0.7	5.6 ± 0.3	0.426
CRP (mg/L)	2.4 ± 0.3	2.7 ± 0.4	2.1 ± 0.4	0.383
*Risk factors, n (%)*				
Hypertension	21 (35)	10 (33)	11 (37)	0.637
Hyperlipidemia	31 (52)	13 (43)	18 (60)	0.186
Current smoker	9 (15)	5 (17)	4 (13)	0.511
Family history CAD	2 (3)	0 (0)	2 (7)	0.136
*Medications, n (%)*				
ACEi/ARBs	19 (32)	9 (30)	10 (33)	0.643
CCB	5 (8)	3 (10)	2 (7)	0.728
β-Blockers	10 (17)	4 (13)	6 (20)	0.415
Diuretics	8 (13)	5 (17)	3 (10)	0.266
Statins	30 (50)	13 (43)	17 (57)	0.186
Fibrates	5 (8)	3 (10)	2 (7)	0.698

Data are expressed as the mean ± standard deviation or number (%). CoQ10, coenzyme Q10; HbA1c, glycosylated hemoglobin; CRP, C-reactive protein; CAD, coronary artery disease; ACEi, angiotensin-converting enzyme inhibitors; ARBs, angiotensin receptor blockers; CCB, calcium channel blockers

### Effect of CoQ10 in metabolic parameters

At the 6-month follow-up, compared to baseline, patients who received CoQ10 had significantly improved plasma LDL levels (102 ± 21 mg/dL vs. 113 ± 27 mg/dL, *p* = 0.045; Table 2), while changes in the placebo group at the 6-month follow-up, compared to baseline, were statistically insignificant (101 ± 34 mg/dL vs. 108 ± 32 mg/dL, *p* = 0.085). Νo significant differences were observed in BMI, TC, HDL and TG levels, HbA1c and CRP among the study groups throughout the follow-up period (*p* > 0.05; Table 2).

**Table 2:** Changes in metabolic parameters in the study population during the study period

	CoQ10 (*n* = 30)			Placebo (*n* = 30)		
	Baseline	6 months	*p*-value	Baseline	6 months	*p*-value
BMI (kg/m^2^)	32 ± 5.8	31.5 ± 5.2	0.125	31.4 ± 4.4	31 ± 4.6	0.108
TC (mg/dL)	188 ± 32	182 ± 25	0.209	177 ± 29	178 ± 35	0.583
HDL-C (mg/dL)	51 ± 12	50 ± 11	0.199	50 ± 11	49 ± 12	0.495
LDL-C (mg/dL)	113 ± 27	102 ± 21	**0.045**	108 ± 32	101 ± 34	0.085
TG(mg/dL)	131 ± 65	133 ± 48	0.810	130 ± 53	128 ± 49	0.888

Bold value denotes statistical significance at the *p* < 0.05 level

Data are presented as mean ± standard deviation. CoQ10, coenzyme Q10; BMI, body mass index; TC, total cholesterol; HDL-C, high-density lipoprotein cholesterol; LDL-C, low-density lipoprotein cholesterol; TG, triglycerides

### Effect of CoQ10 in liver steatosis and liver fibrosis

At the 6-month follow-up, compared to baseline, patients who received CoQ10 had significantly improved Liver Steatosis (280.9 ± 33.4 dB/m vs. 304.8 ± 37.4 dB/m, *p* = 0.017; Table 3) while changes in the placebo group were statistically insignificant (282 ± 34.5 dB/m vs. 290.8 ± 39.7 dB/m, *p* = 0.437). Νo significant differences were observed in Liver Fibrosis among the study groups throughout the follow-up period (*p* > 0.05; Table 3).

**Table 3:** Changes in liver steatosis, liver fibrosis, vascular, endothelial and myocardial markers in the study population during the study period

	CoQ10 (*n* = 30)			Placebo (*n* = 30)		
	Baseline	6 months	*p*-value	Baseline	6 months	*p*-value
CAP Score (dB/m)	304.8 ± 37.4	280.9 ± 33.4	**0.017**	290.8 ± 39.7	282 ± 34.5	0.437
E (kPa)	6.5 ± 3.4	6.1 ± 3.5	0.562	6.6 ± 4.3	6.2 ± 3.1	0.461
PBR (5–25 μm)	2.29 ± 0.18	2.18 ± 0.23	**0.035**	2.26 ± 0.22	2.25 ± 0.18	0.994
SBP (mmHg)	135 ± 13	128 ± 12*	**0.006**	133 ± 16	134 ± 14*	0.682
DBP (mmHg)	84 ± 6	83 ± 11	0.886	82 ± 7	83 ± 8	0.461
Central SBP (mmHg)	128 ± 17	121 ± 15*	**0.049**	125 ± 13	126 ± 15*	0.747
AIx (%)	18.5 (1.3–31.2)	8.2 (−12.4–25.5) *	**0.032**	19.1 (1.3–35.1)	16.9 (3.2–27.4)*	0.770
PWV (m/s)	10.2 ± 2.3	9.5 ± 2*	**0.018**	10.7 ± 3	10.8 ± 2.8*	0.935
FMD	4.3 ± 2.8	6.1 ± 3.8	**0.002**	4.5 ± 3.3	5.1 ± 3.7	0.312
CFR	2.8 ± 0.4	3.1 ± 0.4	**0.014**	2.8 ± 0.4	2.95 ± 0.3	0.133
GLS (%)	−18.8 ± 1.9	-19.6 ± 1.6	**0.011**	−19.2 ± 2.5	-19.2 ± 2.1	0.913

Bold values denote statistical significance at the *p* < 0.05 level

Data are presented as mean ± standard deviation or median (first quartile–third quartile). ANOVA for repeated measurements was applied for comparisons between groups. The model was adjusted for age, sex, body mass index, hypertension, hyperlipidemia, smoking and concomitant medical treatments. CoQ10, coenzyme Q10; CAP, controlled attenuation parameter; E, fibrosis score), PBR, perfused boundary region; SBP, systolic blood pressure; DBP, diastolic blood pressure; AIx, augmentation index; PWV, pulse wave velocity; FMD, flow-mediated dilation, CFR, coronary flow reserve; GLS, global longitudinal strain. * *p* < 0.05 for comparisons of CoQ10 vs. placebo after treatment

### Effect of CoQ10 in vascular and endothelial function

At the 6-month follow-up, compared to baseline, treatment with CoQ10 resulted in significant reduction of PBR of sublingual artery microvessels (2.18 ± 0.23 vs. 2.29 ± 0.18 μm, *p* = 0.035) brachial Systolic Blood Pressure (SBP) (128 ± 12 vs. 135 ± 13 mmHg, *p* = 0.006) central SBP (121 ± 15 vs. 128 ± 17mmHg,, *p* = 0.049)and Aix (8.2% vs. 18.5%, *p* = 0.032 (Table 3). Furthermore, at the 6-month follow-up, compared to baseline, patients receiving CoQ10 showed significant improvement in arterial elasticity as assessed by PWV (9.5 ± 2 vs. 10.2 ± 2.3 m/sec, *p* = 0.018) and FMD (6.1 ± 3.8 vs. 4.3 ± 2.8%, *p* = 0.002)(Table 3), while no changes were observed in placebo group in all above parameters(*p* > 0.05for all measurements; Table 3). At 6 months, statistically significant differences were observed between CoQ10 and placebo in SBP (128 ± 12 mmHg vs. 134 ± 14 mmHg, *p* = 0.045), central SBP (121 ± 15 mmHg vs. 126 ± 15 mmHg, *p* = 0.037), AIx (8.2% vs. 16.9%, *p* = 0.022) and PWV (9.5 ± 2 m/s vs. 10.8 ± 2.8 m/s, *p* = 0.040).

### Effect of CoQ10 in myocardial function

At the 6-month follow-up, compared to baseline, patients of CoQ10 group showed significant improvement in Coronary flow reserve (3.1 ± 0.4 vs. 2.8 ± 0.4, *p* = 0.014) and global longitudinal strain of the Left Ventricle(-19.6 ± 1.6% vs. -18.8 ± 1.9%, *p* = 0.011). Placebo group changes on those parameters were not significant (*p* > 0.05 for all measurements; Table 3).

### Association of liver steatosis with endothelial, vascular and LV myocardial function

>In the whole study population, CAP score was directly associated to PBR 5–25 μm (*r* = 0.31, *p* = 0.028), PWV (*r* = 0.40, *p* = 0.024) and GLS (*r* = 0.28, *p* = 0.041) and inversely correlated to CFR (*r* = -0.29, *p* = 0.032). Additionally, increased PBR 5–25 μm was associated with increased PWV (*r* = 0.35, *p* = 0.041) and reduced FMD (*r* = -0.37, *p* = 0.039).

**Fig. 2:**
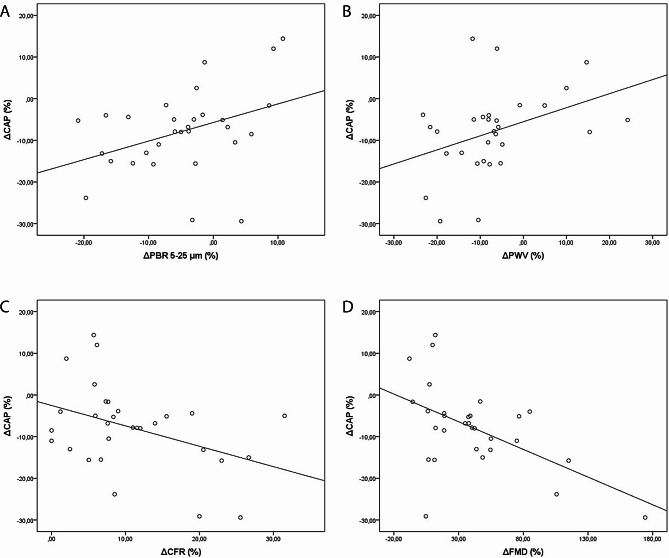
Controlled attenuation parameter **(**CAP) score changes and their correlation with perfused boundary region (PBR) [part A], pulse wave velocity (PWV) [part B], coronary flow reserve (CFR) [part C] and flow-mediated dilation (FMD) [part D] changes in coenzyme Q10 study population

In patients under CoQ10, the reduction in CAP score was positively related to the reduction in PBR 5–25 μm (*r* = 0.38, *p* = 0.038) and in PWV (*r* = 0.39, *p* = 0.036) and reversely related to the increase in CFR (*r* = -0.35, *p* = 0.042) and in FMD (*r* = -0.43, *p* = 0.017) (Fig. 2).

### Adverse events

No serious adverse events were recorded during study period. Two participants discontinued the administration of the investigational drug during the study; in the placebo group one participant reported experiencing lightheadedness, while in the CoQ10 group a participant experienced mild pain in the right upper abdominal quadrant which was successfully alleviated with proton-pump inhibitors. Additionally, one participant from the CoQ10 group was lost to follow-up due to a change of hometown.

## Discussion

The global prevalence of MASLD is on the rise, and its association with cardiovascular events is substantiated by baseline endothelial, vascular, and myocardial dysfunction. Notably, there is a dearth of efficacious pharmaceutical interventions for the treatment of MASLD.

In this randomized, prospective trial involving patients with MASLD, daily coenzyme Q10 at a dose of 240 mg led to larger reductions in liver steatosis compared to the placebo group at the 6 months follow-up. In addition, CoQ10 resulted in remarkable improvement of endothelial, vascular and myocardial function compared to the placebo group. Meaningful improvement in the lipid profile were more common in the CoQ10 group than the placebo group. Moreover, treatment with CoQ10 led to similar adverse events with placebo and no drug discontinuation due to serious adverse events.

In a recent meta-analysis with NAFLD patients, the effect of CoQ10 supplementation on lipid profile revealed non-significant decrease in TC, LDL, HDL, and TG [28]. In the present study individuals who received CoQ10 exhibited significant changes in the plasma levels of LDL signifying substantial improvement in their lipid profile. It is crucial to note that the duration of the intervention may be a key factor in determining the effectiveness of CoQ10 supplementation, since in the studies included in the meta-analysis, treatment spanned from 4 to 12 weeks, whereas in the present study, treatment extended to 24 weeks. Furthermore, it should be noted that patients with NAFLD might exhibit diverse responses to distinct CoQ10 dosages. In above studies CoQ10 dosage varied from 20 to 100 mg daily, while in the present study patients received 240 mg per day. Comparative studies with different CoQ10 dosages are of utmost importance to determine their varying effects on lipid profile and liver steatosis. Similar to the present study, combination therapy involving antioxidants, vitamins, and herbs has demonstrated potential efficacy in other studies [29].

In our study, all participants received comprehensive dietary guidance that adhered to the fundamental principles of the Mediterranean diet. Additionally, they were motivated and encouraged to actively engage in a minimum of 150 min of moderate exercise on a weekly basis. The dietary instructions aimed to align with the holistic approach of the Mediterranean diet, emphasizing the consumption of nutrient-rich foods, while the encouragement for regular physical activity underscored the importance of maintaining a healthy and active lifestyle. The diminished CAP score observed in both groups encompasses all the mentioned factors above; however, individuals who received CoQ10 exhibited significant improvement in liver steatosis, despite the BMI remaining constant. This indicates significant improvement in hepatic fat accumulation.

This study is the second one to report liver steatosis changes with CoQ10 supplementation. Farsi et al. [30] conducted a randomized study involving 41 NAFLD patients, who were commenced on either 100 mg of CoQ10 or placebo daily for 3 months. Liver aminotransferases, inflammatory biomarkers and NAFLD grades were evaluated and significantly decreased, indicating the positive effect of CoQ10 on NAFLD. In our study, we analyzed a larger cohort of patients over a longer period of time and supplemented them with 240 mg of CoQ10.Unlike Farsi et al., who assessed NAFLD grades using ultrasonography, we employed Fibroscan, a method that provides a more objective and quantitative evaluation of steatotic liver disease. A key distinction between our study and that of Farsi et al. is our focus on the endothelial, vascular, and myocardial effects of CoQ10, rather than solely on liver biochemistry and inflammation markers. Nevertheless, both studies revealed a positive impact of CoQ10 with minimal adverse events over a relatively long period of time.

CoQ10 has been shown to lower blood pressure at doses between 100 and 200 mg per day in a number of studies involving human participants [31, 32]. In the course of the present study, the administration of CoQ10 led to a noteworthy decrease in systolic blood pressure, registering a reduction of 7 mmHg, while its impact on diastolic blood pressure was observed to be minimal. These findings indicate a potential role for CoQ10 in managing hypertension, whether used independently or in combination with other conventional anti-hypertensive medications. However, it is imperative to conduct more meticulously monitored clinical trials.

Endothelial dysfunction is a condition that contributes to the pathophysiology of vascular disease in MASLD patients. It manifests prior to the development of atherosclerotic lesions or the occurrence of clinical events and is considered as a trustworthy marker of subclinical atherosclerosis. Endothelial function improvements with CoQ10 supplementation have been shown mainly in patients with DM with 2 clinical trials using 200 mg/day of CoQ10 for 12 weeks showing significant improvements in brachial artery flow-mediated dilatation [33, 34]. Larijiani et al. [35] supplemented 65 intermediate risk individuals with 30 mg/day CoQ10 for 1 year and showed significant improvements in PWV. In our study, we assessed endothelial function using PBR, a technologically advanced and sophisticated method. We demonstrated, six months post-treatment with CoQ10, a substantial reduction in the PBR of sublingual artery microvessels and a decrease in Aix. Additionally, patients receiving CoQ10 exhibited significant alterations in arterial elasticity, as evidenced by changes in PWV and FMD of the brachial artery. It is crucial to highlight that these impactful changes were absent in the placebo group across all the aforementioned vascular metrics. This comprehensive analysis underscores the multifaceted positive effects of CoQ10 on vascular health. The observed reductions in PBR and AIx indicate improved microvascular perfusion and reduced arterial stiffness, respectively. The notable changes in PWV and FMD further signify enhanced arterial flexibility and endothelial function in patients undergoing CoQ10 treatment. These findings not only contribute to the growing body of evidence supporting the cardiovascular benefits of CoQ10 but also emphasize its potential role in ameliorating various aspects of vascular function.

Administering CoQ10 may also be beneficial as adjunctive treatment in heart failure. CoQ10 has been demonstrated to ameliorate symptoms, lower adverse cardiovascular events, and lower cardiovascular mortality in individuals with heart failure [36, 37]. Considering the heightened cardiovascular risk associated with MASLD patients, this study demonstrated a noteworthy correlation between CoQ10 supplementation and left ventricular function. In particular, we observed a significant improvement in coronary microcirculation, as assessed by CFR, and an improvement in left ventricular performance as assessed by GLS.

A major finding in our study was the notable association between the percentage reduction in CAP score and improvements in PBR and PWV. This suggests that as the CAP score decreased, indicative of reduced liver steatosis, there were concurrent positive changes in PBR and PWV. Additionally, the study revealed a reverse relationship between the percentage reduction in CAP score and the percentage increase in CFR and FMD of the brachial artery, further highlighting the potential cardiovascular benefits associated with the reduction in liver steatosis in the presence of CoQ10 supplementation. These findings underscore the complex interplay between liver health and cardiovascular function, shedding light on the potential role of CoQ10 in ameliorating liver steatosis and its correlated improvements in endothelial, vascular, and myocardial aspects.

This study has some limitations. It is a single center study with a relatively small number of participants. Nevertheless, it thoroughly evaluates the effect of CoQ10 in a well selected population with MASLD, matched for baseline characteristics, by studying endothelial, vascular and myocardial function. In order to better understanding the effects of CoQ10 administration, prospective large-scale studies with longer follow-up are needed.

### **Conclusions**

In conclusion, six-month treatment with high doseCoQ10 in patients with MASLD resulted in reduced liver steatosis in parallel with an improvement in endothelial, vascular and myocardial function compared to placebo. Thus, CoQ10 administration may confer significant reduction of the cardiovascular risk in patients with MASLD. Larger scale studies with longer follow-up are needed to fully comprehend the CoQ10 effects in this specific population.

## Data Availability

The datasets used and/or analysed during the current study are available from the corresponding author on reasonable request.

## Abbreviations

MASLD: Metabolic Dysfunction Associated Steatotic Liver Disease
CoQ10: Coenzyme Q10
ROS: Reactive Oxygen Species
CVD: Cardiovascular Disease
NAFLD: Non-alcoholic Fatty Liver Disease
DM: Diabetes Mellitus
HDL: High Density Lipoprotein
BMI: Body Mass Index
HbA1c: Glycated Hemoglobin
TC: Total Cholesterol
TG: Triglycerides
LDL: Low-Density Lipoprotein
CRP: C-reactive Protein
OGTT: Oral Glucose Tolerance Test
CAP: Controlled Attenuation Parameter
LV: Left Ventricular
GLS: Global Longitudinal Strain
PWV: Pulse Wave Velocity
FMD: Flow Mediated Dilation
CFR: Coronary Flow Reserve
S: Steatosis
PBR: Perfused Boundary Region
AIx: Augmentation Index
SD: Standard Deviation
ANOVA: Analysis of Variance
SBP: Systolic Blood Pressure
